# Hypothesis paper: electroacupuncture targeting the gut–brain axis to modulate neurocognitive determinants of eating behavior—toward a proof of concept in the obese minipig model

**DOI:** 10.1007/s40519-020-00864-0

**Published:** 2020-02-25

**Authors:** Xuwen Zhang, Hanwei Chen, David Val-Laillet

**Affiliations:** 1 Panyu Central Hospital, Guangzhou, China; 2 Guanzhou University of Chinese Medicine, Guangzhou, China; 3 INRAE, INSERM, Univ Rennes, Nutrition Metabolisms and Cancer, NuMeCan, 16 Le Clos, St Gilles, 35590 Rennes, France

**Keywords:** Obesity, Vagus nerve, Brain activity, Brain imaging, Pig model

## Abstract

Acupuncture has thousands of years of history and perspective for the treatment of many health problems and disorders. Beneficial effects of acupuncture on obesity have been demonstrated at various levels in animals and clinical trials, with almost no adverse effect, even when combined with local electrical stimulation, i.e., electroacupuncture (EA), a way to potentiate the effects of acupuncture. However, there is still scattered evidence about the impact of EA on brain functions related to the control of eating behavior, and notably on the gut–brain axis mechanisms involved in these putative central modulations. During the past 10 years, we have described a convincing diet-induced obese minipig model, and successfully implemented brain imaging and neurocognitive approaches to challenge mechanistic hypotheses and innovative therapeutic strategies. In the present article, we propose to confront the current literature on the acupuncture and EA effects on the gut–brain axis and obesity with the latest developments in nutrition and neuroscience research using the minipig model. Our aims are to (a) elaborate functional hypotheses on the gut–brain mechanisms underlying EA effects on obesity, and especially on the role of the vagus nerve, and (b) present the rational for testing these hypotheses in the minipig model.

## Introduction

**Fig. 1:**
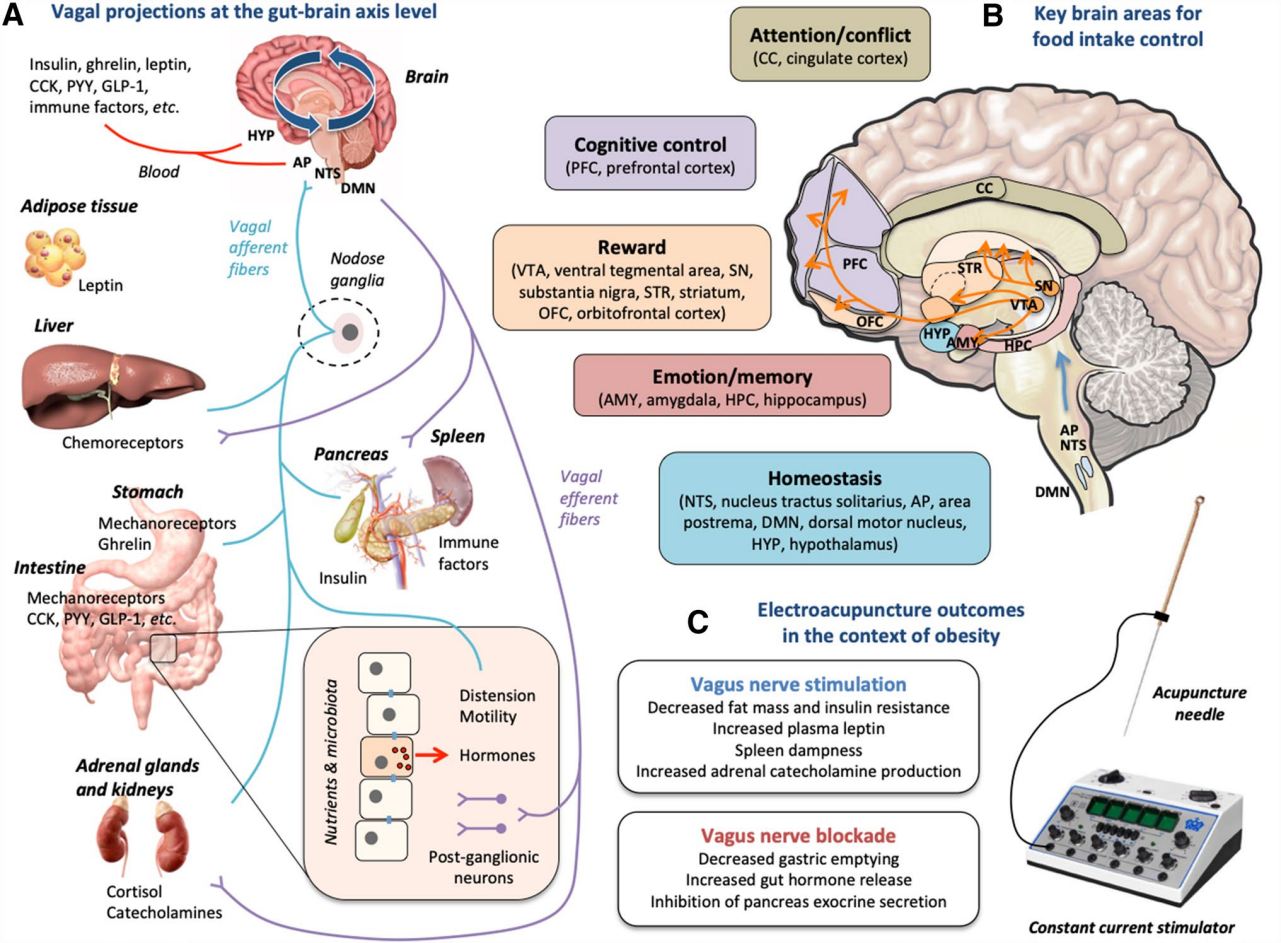
**a** Schematic representation of the vagal afferent and efferent projections at the gut–brain axis level. Peripheral signals reach the solitary tract and area postrema complex (NST/AP) and/or hypothalamus via afferent vagal fibers (blue lines) and/or blood (e.g., orexigenic or anorectic hormones, pro- or anti-inflammatory factors, etc.). Vagal afferents neurons, located in the nodose ganglia, express chemoreceptors on their terminals in the gut that sense these hormones, as well as mechanoreceptors that sense distension. The NTS projects to the dorsal motor nucleus (DMN), which modulates in return, via efferent vagal fibers (purple lines), the intestinal motility and secretion, glucose production, and pancreatic and adrenal glands endocrine activity. Adapted from Guarino et al. [99] and de Lartigue [100]. **b** Schematic representation illustrating key brain areas involved in homeostasis, emotion and memory, hedonism and reward, cognitive control, as well as attention and cognitive conflict. Homeostatic, hedonic, limbic, and cognitive circuits regulating food intake are interconnected and modulated by nervous and hormonal food signals. Dopaminergic projections (orange lines) are indicated as an example of neurochemical modulation, since chronic impairment of vagus nerve functions can inhibit brain dopamine neurons, while vagus nerve stimulation can induce dopamine release in the striatum. Adapted from Val-Laillet et al. [11]. **c** Electroacupuncture performed on specific acupoints is hypothesized to modulate the vagosympathetic balance, the bilateral gut–brain communication, as well as the activity of brain areas involved in food intake control. Electroacupuncture can induce vagus nerve stimulation or blockade, with specific peripheral outcomes described in the context of obesity. For review, see Zhang et al. [13] in the human, and Wang et al. [21] in rodent models. Our main hypothesis is that such outcomes might also contribute to the modulation of brain areas involved in food intake control in obese subjects

Obesity involves a variety of pathogenesis affecting brain functions, gut hormones, the autonomic nervous system, low-grade inflammation and many other biological processes [1–3]. Alteration of the gut–brain communication plays a major role in the emergence of metabolic and behavioral disorders. The importance of the vagus nerve has been well described in these communication processes, and modulation of its activity can modify eating behavior and metabolism. Hormones such as leptin, ghrelin, incretins, or CCK transmit information related to hunger and appetite to the hypothalamus, inhibiting or promoting individual food intake (Fig. 1a). Changes in these hormone levels can impact the homeostatic regulation, but also the brain reward system [4, 5]. Specific neurotransmitters at the central level, such as dopamine, serotonin, and opioids, play a major role in food motivation and hedonism, and these systems can be altered in the context of obesity, therefore impacting food intake control [6–8]. As a consequence, it is hypothesized that an effective and efficient therapeutic treatment should target all these actors of the gut–brain communication and food intake control. It is the case for instance for bariatric surgery, which is the most effective therapy against morbid obesity [9, 10]. Alternative therapies or combination of approaches that would be less invasive, with less secondary effects and more possibilities to individualize the treatment, are still expected. Targeting the gut–brain axis using neuromodulation strategies is a promising research field to fight against obesity and eating disorders [11].

Acupuncture and especially electroacupuncture (EA) are gaining interest in the treatment of obesity, both in humans and animals. In multiple clinical studies, significant outcomes of acupuncture or EA were found on the body mass index, weight loss, waist circumference, fat mass, total cholesterol, and plasma triglycerides [12]. Many researchers hypothesized that these therapeutic strategies modulate complex processes involving the gastrointestinal tract, related hormones, and the central and autonomic nervous systems [12, 13], also inhibiting inflammatory signals [14–16]. EA consists in applying a slight electric stimulation to the acupuncture needles to potentiate the expected effects of acupuncture alone. There is already evidence demonstrating the added value of EA over acupuncture and sham interventions, notably in obese subjects [17]. The effectiveness of acupuncture at abdominal and hindlimb points is related to the activation of afferent nerve fibers [18], and EA is a way to achieve this goal more easily.

In the Traditional Chinese Medicine (TCM) theory, meridians circulate throughout the whole body and communicate with each other. Meridians and acupuncture loci (i.e., acupoints) corresponding to specific organs can be stimulated by acupuncture, with the aim of regulating “qi and blood” as well as some organ functions to treat diseases or alleviate symptoms [16]. Recent researches showed that many acupoints are located in the vicinity or even precise location of nerves bundles, muscle fascia, and blood or lymph vessels. Each acupoint is unique, not only in terms of location but also in terms of associated biophysiological effects. In this paper, we will use the Chinese names to identify the acupoints of interest, and will indicate the World Health Organization (WHO) corresponding codes in brackets. In pig acupuncture, specific names and codes are used and we will indicate them in addition to their human equivalents.

Rodents have been widely used in nutrition researches as models of obesity and metabolic disorders [19, 20]. A recent review even described the mechanisms of acupuncture therapy for simple obesity in rodent models, highlighting significant and positive effects on the regulation of lipid metabolism, inflammation, appetite, and browning of white adipose tissue [21]. However, due to the considerable metabolic and physiological differences between humans and rodents, the translation of research findings to humans remains complicated, especially for brain and behavioral research [22]. On the contrary, pigs have a gastrointestinal tract anatomy, morphology, and physiology very comparable to those of humans. Importantly, pigs and humans have most of their cerebral structures in common and their brains appear to be comparable in terms of structure, vascularization, anatomy, growth, and development [23, 24]. Pigs and especially minipigs can serve as an obesity model bridging the gap between rodents and humans [25–27]. In our previous researches at INRA (now INRAE from 1 January 2020), we validated a diet-induced obesity minipig model in terms of behavioral and metabolic responses [28, 29], and described for the first time in an animal model in vivo brain anomalies that were similar to those observed in obese humans [30]. As a consequence, the pig model represents an excellent opportunity to investigate the impact of acupuncture or EA on the gut–brain determinants of obesity.

In this hypothesis paper, we will first summarize the state of the art supporting an action of EA on the nervous and hormonal pathways connecting the gut to the brain, describing how modulation of the gut–brain communication can influence the central processes underlying food intake control. Second, in light of our 10-year experience in nutrition and neuroscience research in the minipig model at INRA [31], we will justify the implementation of EA in this model to investigate its outcomes in the context of obesity.

## Regulation of brain responses and eating behavior by gut–brain signals: the major role of the vagus nerve

Eating behavior is regulated by several systems or functions in the brain (Fig. 1b), including the homeostatic control system (e.g., medulla, hypothalamus), the reward network (e.g., ventral tegmental area, striatum, orbitofrontal cortex), and the cognitive control system (e.g., prefrontal cortex, cingulate cortex) [32]. The role of homeostasis is to balance physiological parameters by controlling food intake and energy expenditure according to the metabolic needs. The hedonic dimension of food intake sometimes overrules this regulation system, leading to food overconsumption and loss of control. This is frequently the case in the context of obesity and specific eating disorders, such as food cravings and addictions [11]. The cognitive control allows people to decline a piece of cake when they are hungry, or to prefer less palatable food items if they are healthier [33]. Compared to lean subjects, obese men have less activation in the dorsolateral prefrontal cortex, a brain area implicated in the inhibition of inappropriate behavior, satiety, and meal termination [11]. In our diet-induced obese adult minipigs, similar deactivations were observed in the dorsolateral and anterior prefrontal cortices, as well as in the ventral striatum, compared with normal-weight minipigs [30]. Moreover, the anterior and dorsolateral prefrontal cortices as well as the insular cortex activity was negatively associated with the body weight [30].

The vagus afferent pathway is probably the most important link between the gut and the brain [34]. The afferent components of the vagus nerve transmit information from the gastrointestinal tract to the solitary nucleus and communicate with the efferent components of the dorsal motor nucleus of vagus (DMV) and other nuclei returning to the target organs, the vagus nerve acting as a bridge [35, 36]. Gut hormones such as ghrelin, incretins, or CCK have the ability to bind to specialized receptors on the vagus nerve and trigger afferent signals (Fig. 1a). In combination with central dopamine for example, they transmit information about the meal caloric density and composition, modulating the alternation of appetite and hunger in the brain, as well as the reward system and hedonic motivation. The disturbance of this gut–brain communication can favor the onset of weight gain and eating disorders such as food addictions [4, 5, 37]. In humans, the obesity-related functional brain anomalies described in the prefrontal cortex and striatum are often associated with a depletion of the dopaminergic system, which underpins the “reward deficit theory” [38, 39]. Interestingly, adult Yucatan minipigs that were exposed to sugar and fat in the young age through the maternal diet showed lower dopamine transporter binding potential in several brain areas as well as lower cognitive abilities during the alley maze test [40]. Deleterious nutrition in the minipig model is consequently a potent trigger for brain anomalies that are comparable to what is described in obese humans.

**Fig. 2:**
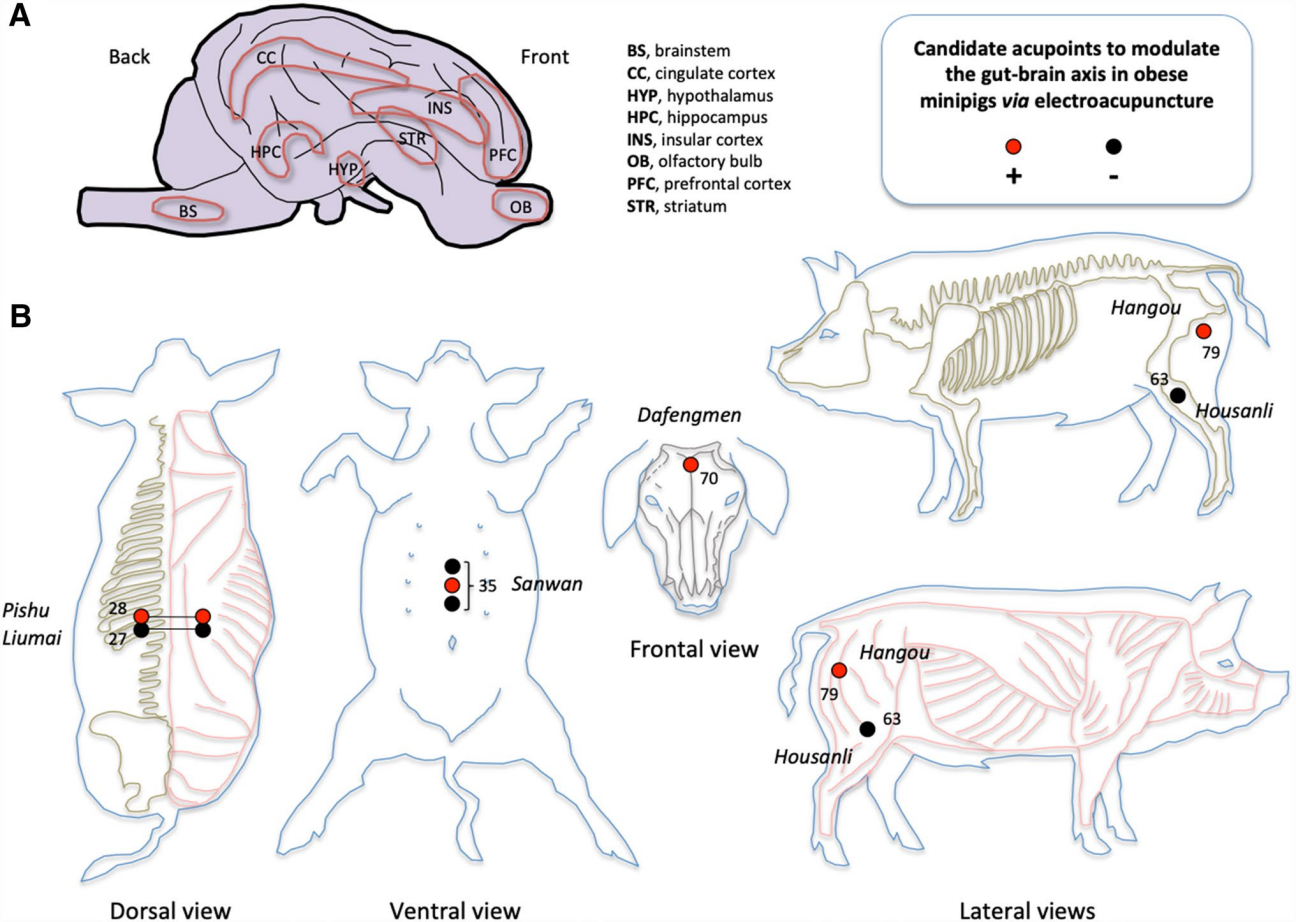
Implementation of electroacupuncture (EA) in the pig model to modulate brain areas involved in food intake control and metabolism. **a** Pig brain schematic representation illustrating the brain areas that were found modulated with vagus nerve stimulation in obese minipigs, together with decreased food intake and weight gain, and improved insulin sensitivity. Adapted from Val-Laillet [31]. **b** Pig acupuncture charts illustrating the location of candidate acupoints (Pishu (#28) and the third pair of Liumai (#27); Sanwan (#35) and Dafengmen (#70); Hangou (#79) and Housanli (#63)) to modulate the gut–brain axis communication, metabolism, brain activity and eating behavior in diet-induced obese minipigs. Stimulating these acupoints is hypothesized to modulate the vagosympathetic balance and related gut–brain communication pathways, as well as the central processes presented in Fig. 1. Acupuncture charts were adapted from the textbook “Traditional Chinese veterinary acupuncture and moxibustion” [81]. Detailed information about the anatomical position and local innervation of the acupoints of interest are provided in Table 1

Previous research in the pig model showed that chronic abdominal vagal stimulation reduced food intake as a consequence of the activation of the brainstem and higher-order brain areas (prefrontal cortex, thalamus, insular cortex, superior colliculus, cingulate cortex) [41, 42] (Fig. 2a). In obese minipigs, this treatment was also associated with reduced weight gain and preference for a sweet feed [43]. Chronic vagal stimulation was also associated with increased glucose metabolism in the cingulate and prefrontal brain areas (Fig. 2a), and substantially improved insulin sensitivity in diet-induced obesity minipigs, via both peripheral and central mechanisms [42]. One of the hypotheses presented in the current paper is that it is possible to mimic the VNS-induced effects using electroacupuncture on specific acupoints in minipigs as well as in humans.

## Impact of EA on the vagus nerve and other actors of the autonomic nervous system (ANS)

Among several biological actors that may contribute to the acupuncture’s mode of action, the autonomic nervous system (ANS) is a good candidate because it can interconnect external somatosensory inputs with internal organ responses via the central nervous system [44, 45]. Of the sympathetic and the parasympathetic nerves composing the ANS, the vagus nerve, which broadly regulates the functions of internal organs, has been a primary target for exploring the possible effect of acupuncture stimulation on internal organs [18, 46–48].

In healthy subjects, the sham-feeding-stimulated vagal gastric acid secretion was significantly reduced by EA at ST36, BL21, and transcutaneous electric nerve stimulation, but not by classic acupuncture or laser acupuncture [46]. The stimulation of gastric acid secretion by sham feeding entails mainly the central vagal component of gastric acid stimulation [49]. Lux et al. [46] suggested that EA might reduce gastric acid secretion by means of afferent visceral fibers that are related with the vagal nerve system. In addition, in a mouse model of polymicrobial peritonitis, EA at Zusanli (ST36) controlled systemic inflammation by inducing vagal activation of aromatic l-amino acid (DOPA) decarboxylase, leading to the production of dopamine in adrenal medulla. The authors suggested the existence of a novel EA-induced anti-inflammatory mechanism mediated by the sciatic and the vagus nerves, modulating the production of catecholamines in the adrenal glands (Fig. 1a), and which could be mimicked pharmacologically by selective dopamine agonists [48].

Other evidences support the idea that acupuncture or EA can modulate the vagosympathetic balance. In healthy human volunteer subjects, acupuncture stimulation at Ximen (PC4) decreased heart rate [47], an effect that was attenuated by the administration of atropine and propranolol. Therefore, the acupuncture-induced response of a decrease in heart rate was concluded to be a result of a reciprocal coordination of an increase in cardiac vagal activity and a decrease in cardiac sympathetic activity [47]. In animal experimentations using acupuncture, the subjects are very often anesthetized to facilitate handling and the acupuncture treatment, while reducing and homogenizing the individual reactivity. Interestingly, Sato et al. [50] demonstrated in anesthetized animals that cardiac vagal activity is specially depressed by anesthesia, contrary to gastric vagal activity. This point is very important for the scientific demonstration that we aim for in the minipig model, because, to modulate the gut–brain communication, these are the gastric vagal afferent which are targeted. In pigs, Takamiya et al. [51] demonstrated that EA on the limbs significantly suppressed cardiac excitability during left stellate ganglion stimulation through correction of the vagosympathetic balance (attenuation of the increase in LF/HF).

In contrast, other investigations provided evidences that different types of nerve fibers can transmit EA stimulation at the location of body trunk and hindlimb. Noguchi [52] summarized several studies in anesthetized rats (or dogs) showing that EA performed on abdominal acupoints can inhibit gastric motility by exciting the efferent fibers of the gastric sympathetic nerves. On the contrary, EA performed on the hindlimb, at Zusanli (ST36) for example, managed to increase motility by exciting the efferent fibers of the gastric vagus nerve. Comparable effects were observed for duodenal responses and a complex relationship between EA intensities and the motility changes was described. Depending on the expected effects, different types of fibers might be targeted, such as the group VI fibers of the afferent intercostal nerves in the abdominal area, or the group III fibers of the tibial nerves on the hindlimbs, which justifies calibrating precisely the EA intensity according to the target. Concerning gastric acid secretion, different authors obtained contrasted results, with inhibitory or excitatory effects, which prevented clear explanations about the somatoautonomic and endocrine mechanisms involved [52].

## An example of hormonal factor regulating eating behavior: the role of leptin in gut–brain signaling

The leptin protein is central to the regulation of energy metabolism and eating behavior [53], and has a major role in the obesity etiology. High serum leptin may be an indicator of increased leptin resistance and high risk for obesity, independently from body fat [54]. Leptin might be an afferent signal of the negative feedback loop regulating adipose tissue mass [55]. It affects food/feed intake in humans [56] and pigs [57], and is considered as a signal of the nutritional status modifying the ‘perceived starvation’ during food deprivation periods [58].

The leptin actions on the central nervous system (CNS) play a major role in mediating its metabolic consequences. High levels of leptin receptor mRNA and protein are expressed in the forebrain, especially in the ventromedial hypothalamus, arcuate nucleus (ARC), and dorsomedial areas of the hypothalamus, as well as in the brainstem [59]. Leptin receptor activation of different intracellular signaling cascades in different CNS regions may provide a basis for the phenomenon of selective leptin resistance and divergent regulation of appetite and energy expenditure in the context of obesity [60]. The vagal afferent neurons play an important role in transmitting leptin signaling to the brain. Knockout leptin receptor in sensory neurons of mice increased food intake, meal size, and meal duration; furthermore, CCK- and leptin-induced satiation were reduced [61]. Leptin potentiates the post-prandial release of gastrointestinal hormones such as CCK, PYY, and GLP-1, which activate vagal afferent neurons to mediate satiation control and meal termination [62]. Leptin signaling in vagal afferent neurons is required for CCK-induced satiation [61].

## EA as leverage to modify the gut–brain communication through modulation of the vagosympathetic balance and hormonal factors

As previously stated, direct bilateral vagal stimulation in the obese minipig was successful in activating the DVC and corticolimbic brain structures, reducing food intake and weight gain, as well as improving insulin sensitivity via peripheral and central mechanisms [41–43]. But non-invasive approaches to stimulate the vagus nerve have also been successfully tested. Kraus et al. [63] performed electrical stimulation of the nerves in the left outer auditory canal of healthy subjects, with the aim of activating vagal afferences transcutaneously (t-VNS). An improvement of well-being assessed with psychometric self-rated scales was observed after t-VNS, and the brain activation patterns observed in these volunteers shared similarities with those observed during invasive vagus nerve stimulation. This result was further confirmed by another fMRI study in humans [64], and in rats multiple t-VNS sessions had antidiabetic effects by regulating glycemia through the triggering of tidal melatonin secretion [65].

Similarly, EA stimulation may generate physiological effects on the autonomic nervous system and especially the vagus nerve, which innervates many internal organs and further triggers brain response (Fig. 1a), with the consequence of regulating metabolic parameters, homeostasis, and eventually eating behavior (Fig. 1b). A few peripheral outcomes of electroacupuncture treatment in the context of obesity are summarized in Fig. 1c. Concerning EA effects at the brain level, the first evidence came from a research demonstrating, through brain molecular biology and immunohistochemistry in rats and mice, that EA at Zusanli (ST36) triggered neuronal responses, including the expression of axonal growth-associated protein in the dorsal root ganglia, as well as increased c-Fos transcription factor in the motor nucleus of the vagus nerve (DMV) and nucleus tractus solitarius (NTS) [66]. The DMV and NTS play a major role in integrating visceral somatosensory signals, notably through the vagus nerve and solitary tract, and generating feedback to the splanchnic area [67]. Wang et al. [68] demonstrated in rats that EA at Zhongwan (RN12) and Weishu (BL21) induced neuronal activation in the dorsal vagal complex (DVC) and paraventricular hypothalamic nucleus (PVN), increased the levels of gastrin and gastrin receptors in the gastric antrum and PVN, and regulated gastric motility. A more recent study demonstrated in a mouse model of lipopolysaccharide (LPS)-induced acute inflammation that acupuncture at Zusanli (ST36), via the transmission of signals through the vagus nerve and the activation of the DVC, attenuated the inflammatory response assessed by TNF-α expression in the serum and spleen [69]. A comparable experiment in rats showed that EA at Zusanli (ST36) on the hindlimb, in comparison to Shousanli (LI10) on the forelimb, increased gastric acid secretion [70]. This response was abolished after sciatic denervation or vagotomy, but enhanced after sympathectomy. The authors concluded that the EA effects were dependent on the somatic nerves for the afferent pathway, as well as the vagus nerve to the stomach for the efferent pathway [70]. Other factors than somatoautonomic reflexes might be involved in this regulation process, such as hormonal and emotional factors [18].

Hormonal factors such as gut hormones leptin, ghrelin or dopamine may play a role as mentioned above. Acupuncture treatment for obesity had beneficial effect on serum leptin levels compared to no treatment or oral anorectic drug, and this effect was even increased when combined with diet therapy or exercise [71]. Five-week acupuncture treatment showed beneficial effects on insulin, leptin, ghrelin, and CCK levels in obese women compared to sham (non-penetrating) acupuncture group, even after a few weeks of treatment [72]. Compared with a food-restricted group, the EA group showed a significant body weight loss and decrease in serum leptin levels in women [73]. Finally, a clinical trial on obesity and overweight in human volunteers in Iran demonstrated a greater reduction of plasma leptin in the EA group than in the sham group [74]. Ghrelin is known to target the hypothalamus (HYP) for regulating eating behavior [75]. The roles of ghrelin feedback on the appetite regulating network are crucial for energy homeostasis and appetite [76]. Evidence suggested that the orexigenic peptides, including ghrelin and neuropeptide Y (NPY), could be down-regulated by EA, therefore decreasing food intake in rats [77]. Even more interesting is the work performed by von Deneen et al. [78] who found in fasting overweight male volunteers that different functional neural networks correlated with EA-induced effects on blood glucose, core body temperature, and hunger, respectively. The increased dopamine (DA) during acute acupuncture at Zusanli (ST36) and Yinlingquan (SP9) was probably associated with modulations of the poststimulation limbic system and spinothalamic tract connectivity, with positive or negative correlations between acupuncture-induced changes in hunger and two specific networks, HYP-anterior cingulate cortex and HYP-thalamus, respectively [78].

Because of their biophysiological specificities, different acupoints induce different brain responses. In another brain imaging study, Wu et al. [79] found that acupuncture at Hegu (LI4) and Zusanli (ST36) was linked to the activation or deactivation of several limbic system structures. Interestingly, all of these structures are involved in eating behavior and food intake control (Fig. 1b). The hypothalamus plays a role in homeostasis; the nucleus accumbens is part of the reward circuit and was found to be deactivated in obese humans and minipigs [11, 30]; the anterior cingulate cortex is involved in attentional processes and decision-making; while the amygdala and hippocampal complex play a major role in associative learning, emotions, and cognitive processes. For example, the nucleus accumbens and hypothalamus signal activity was increased with acupuncture at Hegu (LI4) and Zusanli (ST36) [79], while the amygdala and hippocampus signal activity was decreased. The existence of different brain responses to acupuncture according to the acupoints selected is not surprising considering that EA stimulation at different body parts cause the activation of different nervous pathways [80]. Further studies need to focus on the specific neurophysiological mechanisms triggered by different combinations of EA stimulations, and their outcomes in terms of brain and behavioral responses.

## State-of-the-art and rationale for implementing acupuncture in the minipig model

The description of acupoints in the pig relies both on anatomical analogies with the human and experimental veterinary science reports. Anatomical charts for acupuncture are available for the pig in veterinary textbooks [81]. Similarly to human acupoints, pig acupoints are located at or near muscle, blood or lymph vessels, or nerves, and have unique biophysiological effects. In veterinary medicine, acupuncture has been used to treat different diseases in pigs [82]. For instance, stimulation of points similar to human Changqiang (GV1), Yaoshu (GV2), Mingmen (GV4) and GV20 (Baihui) is the usual choice to treat impotence and penile paralysis in male boars [83], while acupuncture at Changqiang (GV1), Baihui (GV20), Pishu (BL20) and Zusanli (ST36) produced significant results for treating diarrhea and gut inflammation (for review, [84]). Other authors showed that acupuncture at a locus Dafengmen (#70) similar to the Baihui (GV20) acupoint in the human improved sleep conditions of minipigs [85], demonstrating that stimulation of similar acupoints produces similar therapeutical effects in humans and minipigs. In parallel to human trials, the minipig was also used to test the hypothesis of low hydraulic resistance channels along meridians [86] and demonstrate that blocking these channels trigger a gastric and intestinal distension [87].

Although minipigs share more anatomical similarities with human than rodents, there are still obvious anatomy (e.g., four-legged vs. two-legged) and physiology differences that make impossible to identify in the minipig every acupoint described in the human. It is sometimes tricky to identify in the pig the equivalent locus of a human acupoint. The acupoint Yintang (GV24), for instance, is located between the eyebrows in the human, but there is no equivalent in the pig acupuncture charts. However, Litscher et al. [88] investigated in the pig model whether differences in parameters of bioelectrical brain activity could be found after acupuncture at Yintang (GV24) and Renzhong (GV26). This study revealed a non-significant decrease of the bispectral index with Yintang (GV24) stimulation and an increase with Renzhong (GV26), which is in accordance with studies performed in humans. According to the TCM theory, the acupoint Yintang (GV24) is said to have sedating effects in human medicine, whereas Renzhong (GV26) is said to have stimulating effects.

All these studies demonstrate that, to implement acupuncture in pigs for research and veterinary purposes, it is necessary to combine the fundamental principles of the TCM theory with anatomical/physiological analogies between species, as well as existing veterinary reports confirming significant outcomes.

## Toward a preclinical study in the diet-induced obese minipig to modulate the gut–brain axis and eating behavior

On the basis of the aforementioned state of the art, we propose implementing EA in the diet-induced obese minipig model to identify the best combination of acupoint stimulation to modulate brain areas involved in the different dimensions of food intake control (i.e., homeostasis, hedonism, and executive functions). The ultimate goal is to use EA treatment in obese people to decrease their appetite and food cravings, through the neuromodulation of specific brain areas and processes.

Based on data obtained in the human, rodent and pig models, we selected three sets of acupoint combinations of interest (Table 1; Fig. 2b) including: the pair of pig Pishu (#28) acupoints (anatomically similar to the human Pishu (BL20)) and the third pair of pig Liumai (#27) acupoints (anatomically similar to the human Weishu (BL21)), both located on the back; the three-locus pig Sanwan (#35) acupoint (anatomically similar to the human Shangwan (RN13), Zhongwan (RN12), and Xiawan (RN10)) on the abdomen and pig Dafengmen (#70) on the head (similar to Baihui (GV20) in the human); and a combination of acupoints on the hindlimbs, the pig Housanli (#63) and pig Hangou (#79) (acupoints equivalent of the human Zusanli (ST36) and Huantiao (GB30)). These acupoints were chosen according to the literature previously described in this hypothesis paper as well as to our acupuncturist’s expertise. Concerning Baihui (GV20) on the head (Dafengmen (#70) in the pig), EA at this acupoint demonstrated positive effects in rat models of depression [89] or brain ischemia [90]. Zheng et al. [89] showed that EA at Baihui (GV20) normalized responses in a sucrose-preference test and ameliorated depression-related manifestations by regulating the expression of different genes, notably in the prefrontal cortex. Wen et al. [90] showed improved cognitive abilities and fMRI BOLD brain responses in the hippocampus, cingulate gyrus, and prelimbic cortex. The prefrontal cortex and hippocampus were repeatedly found altered in minipig models of deleterious nutrition and obesity [11, 30, 40, 91], and we hypothesize that regulating their activity via EA at Baihui (GV20), in combination with EA-induced activation of the gut–brain vagal pathway, would contribute to regulate the behavioral and brain processes underlying food intake control, exactly as the more invasive vagus nerve stimulation did in the minipig model [41–43] (Fig. 2a).

**Table 1:** Precise location and anatomical information for the six acupoints selected for our preclinical study in the minipig model

Strategy number	Acupoint name in pigs	Acupoint name in humans	Type of acupoint	Location and anatomical information	Examples of acupuncture and electroacupuncture outcomes	Bibliographic references
1	Liumai (#27)	Weishu (BL21)	Bilateral pair	6 cm lateral to the dorsal midline, in the last three intercostal spaces. Three points on each side. In the muscle groove of *musculi longissimus dorsi* and *iliocostalis dorsi*. Supplied by the intercostal	Reduction of sham-feeding-stimulated vagal gastric acid secretion. Regulation of gastric motility possibly through the PVN–DVC–vagus–gastric neural pathway	[46, 68]
	Pishu (#28)	Pishu (BL20)	Bilateral pair	Corresponding to the second pair of point #27	Treating diarrhea and gut inflammation in pigs	[84]
2	Dafengmen (#70)	Baihui (GV20)	Single	Located on the dorsal mid-sagittal plane between the two ear rostral bases	Positive effects in rat models of depression and brain ischemia. Normalized responses in a sucrose-preference test, ameliorated depression-like symptoms, gene expression regulation in the prefrontal cortex. Improved cognitive abilities and fMRI brain responses. Improved sleep condition in minipigs	[85, 89, 90]
	Sanwan (#35)	Shangwan (RN13), Zhongwan (RN12), and Xiawan (RN10)	3 loci	On the ventral midline of the abdominal region. Middle point is at the midpoint between the caudal end of the xiphoid process of the sternum and umbilicus. Cranial point is at the midpoint between the middle acupoint and the xiphoid process. Caudal point is at the midpoint between the middle acupoint and the umbilicus. Under the skin are *linea alba* and *musculus transversus abdominis*. Supplied by the cranial epigastric, the subcutaneous and the terminal branches of the intercostal	Decreased abdominal pain and distension in human patients with acute pancreatitis. Prevention of nausea and vomiting after pediatric surgery. Regulation of gastric motility possibly through the PVN–DVC–vagus–gastric neural pathway	[68, 101, 102]
3	Housanli (#63)	Zusanli (ST36)	Bilateral pair	In the depression 6 cm caudoventral to the lateral edge of the patella. In the depression ventral to the head of the fibula, in the muscle groove of the long digital extensor and the lateral digital extensor. Supplied by the cranial tibial and the peroneus	Treating diarrhea and gut inflammation in pigs. Modulation of several brain structures in rats, including hypothalamus, hippocampus, nucleus accumbens, and amygdala. Increased dopamine release in adrenal medulla. Increased gut motility by exciting the efferent fibers of the gastric vagus nerve	[48, 52, 66, 84]
	Hangou (#79)	Huantiao (GB30)	Bilateral pair	In the muscle groove between *musculi biceps femoris* and *semitendinosus*, ventral to the ischiatic tuberosity	Reduced anxiety-like behavior and down-regulation of p-ERK in the anterior cingulate cortex in rats	[103]

The six acupoints will be stimulated according to three different combinations, corresponding to three putative therapeutic strategies. Acupoint information came from the textbook “Traditional Chinese veterinary acupuncture and moxibustion” [81]. Examples of acupuncture and electroacupuncture outcomes at these acupoints are indicated with the corresponding references

It is expected that the minipig skin impedance is different from that of humans, which is also different from that of rodents. Each species requires specific methodological adjustments. In obese women, stimulation with a frequency of 5 Hz, wave duration of 1 ms, and intensity of 1.5 mA for 20 min can produce significant effects in terms of weight loss [92]. But even in humans, these parameters must be adjusted according to individual skin impedance or subject’s feeling (e.g., if the subject feels uncomfortable). In anaesthetized minipigs, rather than relying on the individual’s feedback, we will look for objectifiable criteria, such as muscle contraction. This is the case in rats for example, where Luo et al. [15] selected 2-Hz continuous wave for 10 min, with an intensity starting from 1 mA and progressively increasing until the induction of slight muscle contraction. In mice, Choowanthanapakorn et al. [93] used 2-Hz frequency, 100-μs duration, with an intensity of 1 mA for 15 min, whereas Lim et al. [69] selected 1-Hz frequency, 2-ms pulse duration, and 1-V voltage for 30 min. There is still no EA study in minipigs, but the stimulation parameters will be adapted to the model, from 1 mA with progressively increasing intensity until the induction of local muscle contraction. In obese minipigs, the needle insertion depth will be individually adapted according to ultrasonographic measurements of the subcutaneous fat thickness, since most of the acupoints selected (apart from Dafengmen (#70)) target muscle tissues (i.e., below the adiposity layer). According to a recent systematic review and meta-analysis [94], which included 77 studies focusing on electroacupuncture in obesity animal models, 36 trials used continuous waves with frequency of 1–100 Hz, and 14 studies used disperse-dense waves with frequency of 2–100 Hz. The use of continuous wave at 10 Hz was the most commonly used stimulus parameter for electroacupuncture in obese animal models. Our first electroacupuncture tests in normal-weight adult Yucatan minipigs (13-month-old and 35-kg females) demonstrated that a 2-Hz-frequency continuous wave stimulation (KWD-808I electric stimulator; Changzhou Yingdi Electronic Medical devices Co., Ltd.) was sufficient to induce slight muscle contraction on abdominal and hind leg acupoints with an intensity of 2–2.5 mA, as well as on back acupoints with an intensity of 3 mA.

Our preclinical study in the minipig model will mainly aim at describing how EA can neuromodulate specific brain areas and processes involved in food intake, motivation, and pleasure. Basic measurements (previously used in published studies in animal models and/or humans) before, during, and/or after chronic EA treatment will include feed intake and body weight measurements, bio-impedance and ultrasonography measurements to assess body composition, plasma analyses (glucose, gastrointestinal hormones, inflammation marker, cortisol, etc.), hearth rate variability (HRV) via electrocardiography (ECG) measurements to assess the vagosympathetic balance, body thermography measurements to detect any potential increase of the skin temperature during EA, and electroencephalography (EEG) measurements to assess the brain cortical electrical activity. Most important will be the brain functional magnetic resonance imaging (fMRI) sessions performed to map the brain responses to acute EA, as well as the brain responses to oral sugar sensing after chronic EA or sham treatment in obese minipigs. fMRI is a non-invasive neuroimaging approach that can be used to investigate sensory, cognitive, and hedonic integration of exteroceptive or interoceptive stimuli in healthy or pathological subjects. Our laboratory has a world-recognized 10-year experience in using functional brain imaging in the pig model for nutritional and translational research [31]. Notably, we previously described, using fMRI or nuclear brain imaging, the pig brain responses to sucrose stimulation or palatable food flavors [95, 96] and demonstrated that obese minipigs have similar brain anomalies as obese humans [30]. Behavioral correlates will be explored via dedicated behavioral tests that have been extensively implemented, validated, and published, such as food preference and motivation tests, as well as food-rewarded spatial cognitive tests [40, 91].

All the technologies required for this proof of concept and identification of the best EA acupoints combination have already been implemented in the minipig model [31]. As stated before, this encompasses metabolic, physiological, and behavioral explorations, as well as minimally invasive neurobiological measures of the vagus nerve activity [97] and brain responses to various stimuli via fMRI [96, 98]. The fact that significant positive effects of acupuncture and EA in comparison to control treatment in the pig model were demonstrated in several studies indicates the good reproducibility of these approaches in this species [84, 85]. French–Chinese research collaboration was initiated in 2019 to perform this research, of which the aim is to select the best EA strategy, to validate its neurophysiological and neurocognitive effects, and to open the way to a clinical trial in obese human patients.

## What is already known on this subject?

Previous published articles in humans and rodent models demonstrated some beneficial effects of acupuncture and EA in the context of obesity, but further research, notably at the brain and behavioral levels, requires thorough explorations of EA effects in an animal model closer to humans. Rodents have a small and lissencephalic brain, with a general anatomy that is very different from that of humans, which complicates the analogy for acupuncture and neuroscience research. The location of acupoints has been described in pigs. Despite the fact that the minipig model is increasingly used for nutrition and neuroscience research, we found no publication implementing acupuncture or EA in this model and in the context of obesity.

## What does this study add?

The aim of our study is to demonstrate for the first time, in an animal model closer to humans than the usual rodent models, that EA at specific acupoints can modulate the gut–brain axis communication, brain activity, as well as the neurocognitive functions related to food pleasure and motivation. French–Chinese research collaboration was initiated in 2019 to perform this research, of which the aim is to select the best EA strategy, to validate its neurophysiological and neurocognitive effects in terms of food intake control, and to open the way to a clinical trial in obese human patients.
